# Increased Oxidative Stress in Asthma—Relation to Inflammatory Blood and Lung Biomarkers and Airway Remodeling Indices

**DOI:** 10.3390/biomedicines10071499

**Published:** 2022-06-24

**Authors:** Stanisława Bazan-Socha, Krzysztof Wójcik, Magdalena Olchawa, Tadeusz Sarna, Jakub Pięta, Bogdan Jakieła, Jerzy Soja, Krzysztof Okoń, Jacek Zarychta, Lech Zaręba, Michał Stojak, Daniel P. Potaczek, Jan G. Bazan, Magdalena Celińska-Lowenhoff

**Affiliations:** 1 Department of Internal Medicine, Faculty of Medicine, Jagiellonian University Medical College, Skawinska 8, 31-066 Krakow, Poland; krzysztof.wojcik@uj.edu.pl (K.W.); b.jakiela@uj.edu.pl (B.J.); jerzy.soja@uj.edu.pl (J.S.); jzar@mp.pl (J.Z.); magdalena.celinska-lowenhoff@uj.edu.pl (M.C.-L.); 2 Department of Biophysics, Faculty of Biochemistry, Biophysics and Biotechnology, Jagiellonian University, Gronostajowa 7, 30-387 Krakow, Poland; magdalena.olchawa@uj.edu.pl (M.O.); tadeusz.sarna@uj.edu.pl (T.S.); 3 Institute of Applied Radiation Chemistry, Faculty of Chemistry, Lodz University of Technology, Zeromskiego 116, 90-924 Lodz, Poland; jakub.pieta@p.lodz.pl; 4 Department of Pathology, Faculty of Medicine, Jagiellonian University Medical College, Grzegorzecka 16, 31-531 Krakow, Poland; k.okon@uj.edu.pl; 5 Pulmonary Hospital, Gladkie 1, 34-500 Zakopane, Poland; 6 Institute of Computer Science, College of Natural Sciences, University of Rzeszow, Pigonia 1, 35-310 Rzeszow, Poland; lzareba@ur.edu.pl (L.Z.); bazan@ur.edu.pl (J.G.B.); 7 Department of Plant Product Technology and Nutrition Hygiene, Faculty of Food Technology, University of Agriculture in Krakow, Balicka 122, 30-149 Krakow, Poland; michal.stojak@urk.edu.pl; 8 Translational Inflammation Research Division & Core Facility for Single Cell Multiomics, Philipps-University Marburg, 35043 Marburg, Germany; danppot@gmail.com

**Keywords:** asthma, oxidative stress, CBA assay, airway remodeling

## Abstract

Airway inflammation in asthma is related to increased reactive oxygen species generation, potentially leading to tissue injury and subsequent airway remodeling. We evaluated oxidative stress in peripheral blood from asthmatic subjects (*n* = 74) and matched controls (*n* = 65), using recently developed real-time monitoring of the protein hydroperoxide (HP) formation by the coumarin boronic acid (CBA) assay. We also investigated the relation of the systemic oxidative stress response in asthma to disease severity, lung function, airway remodeling indices (lung computed tomography and histology), and blood and bronchoalveolar lavage fluid (BAL) inflammatory biomarkers. We documented enhanced systemic oxidative stress in asthma, reflected by 35% faster and 58% higher cumulative fluorescent product generation in the CBA assay (*p* < 0.001 for both). The dynamics of HP generation correlated inversely with lung function but not with asthma severity or histological measures of airway remodeling. HP generation was associated positively with inflammatory indices in the blood (e.g., C-reactive protein) and BAL (e.g., interleukin [IL]-6, IL-12p70, and neutrophil count). Bronchial obstruction, thicker airway walls, increased BAL IL-6, and citrullinated histone 3 in systemic circulation independently determined increased HP formation. In conclusion, a real-time CBA assay showed increased systemic HP generation in asthma. In addition, it was associated with inflammatory biomarkers, suggesting that proper disease control can also lead to a decrease in oxidative stress.

## 1. Introduction

Asthma is a chronic disease of the airways that is characterized by variable bronchial obstruction and hyperresponsiveness, often accompanied by structural remodeling [1]. Disease pathogenesis involves various cell types and mediators that participate in airway inflammation, trigger asthma symptoms, and contribute to disease progression. Airway inflammation can be exacerbated by viral infections and exposure to inhaled allergens or airway pollutants. Epidemiological studies demonstrate a clear relationship between air quality and control of asthma symptoms. Exposure to tobacco smoke, ozone, and environmental pollution, such as diesel exhaust, generates reactive oxygen species (ROS) and other oxidative stressors, initiating and augmenting inflammation and sensitizing the airways to other triggers of symptoms [1,2]. Additionally, the inflammatory cells present in the asthmatic airways are considered the primary local source of ROS [3]. Furthermore, ROS itself may play a role in asthma pathogenesis, as they promote type-2 (T2) responses in the lungs and activate nuclear factor (NF)-κβ, a potent pro-inflammatory gene inducer [4].

ROS are produced continuously in a small amount by all cells. Still, if delivered in higher amounts, for example, during inflammation, they alter the pro/antioxidant balance, causing oxidative stress and tissue damage [5]. One signature of increased oxidative stress is the hydroperoxides of amino acid residues (HP), unstable derivatives formed during exposure of proteins to ROS [6]. Previous experimental studies confirmed that oxidative damage to proteins, lipids, or nucleic acids might lead to pathological changes in airway epithelial cells, resulting in increased permeability, mucus secretion, and enhanced airway hyperresponsiveness [5,7,8,9].

In asthmatic airways, many cell types enhance ROS production, including epithelial and endothelial cells and infiltrating leukocytes; therefore, ROS are necessary components of the innate immune system [5]. However, the lungs and blood provide an efficient defense system against oxidative stress, mediated by two essential elements. The first contains nonenzymatic dietary antioxidants, including tocopherols, carotenes, and lycopene. The second refers to the endogenous system of antioxidant enzymes, such as superoxide dismutase, catalase, and lipoprotein-associated phospholipase A_2_ (Lp-PLA_2_), which combat biochemically oxidative stress [10]. Despite these mechanisms, asthma patients show increased lung oxidative stress, as evidenced by elevated nitric oxide and carbon monoxide concentrations in the exhaled air [3]. Therefore, it has been suggested that asthma is characterized by a decreased ability to respond to oxidative stress [5,10]. For example, patients with severe asthma show decreased plasma activity of Lp-PLA_2_ [11]. On the contrary, others point to the upregulation of antioxidative mechanisms, albeit with still an overwhelming prooxidative capacity [3,12,13]. Nevertheless, the role of oxidative stress in asthma pathology and airway remodeling has not been comprehensively studied, including how it impacts endothelial injury and early atherosclerosis [14] and increases the risk of prothrombotic and cardiovascular events, as previously reported in that disease [15,16,17].

Numerous studies on asthma indicate an increased prooxidative potential of peripheral blood leukocytes, mainly neutrophils, and upregulation of oxidative biomarkers in airways, e.g., nitric oxide, or in circulation, e.g., malondialdehyde and uric acid [3,5,13,18]. However, scarcer data analyzed oxidative stress globally in circulating blood, probably due to the lack of reliable research methods that could be successfully applied to serum or plasma samples. Current assays are based mainly on the oxidation of ferrous ions, monitored with orange xylenol or the iodometric test [19,20], and are demanding from a technical point of view. Additionally, they cannot be used in real-time measurements. However, recently Michalski et al. [21] developed and validated a novel real-time fluorescent assay that fits this purpose. In this assay, the profluorescent coumarin boronic acid (CBA) probe reacts with amino acid and protein hydroperoxides to form the corresponding fluorescent product, 7-hydroxycoumarin, which is easily detectable by a fluorescent reader.

Considering the available data on the possible link between airway inflammation, local oxidative stress, premature atherosclerosis, and increased risk of cardiovascular events in asthma, we sought to evaluate the CBA assay in the circulating blood of those subjects. We also examined its relation to asthma severity; lung function and morphometry; blood and bronchoalveolar lavage fluid (BAL) inflammatory biomarkers; and histological measures of airway remodeling, including reticular basement membrane (RBM) thickness and collagen I deposits.

To date, such studies have not yet been performed.

## 2. Materials and Methods

### 2.1. Study Participants

We investigated 74 asthma patients enrolled at the Outpatient Clinic of the Allergy and Clinical Immunology Department, University Hospital, Krakow, Poland. Diagnosis of asthma and disease severity (mild, moderate, and severe disease) was established based on the current Global Initiative for Asthma (GINA) guideline [1]. In addition, asthma symptom control was assessed based on the Asthma Control Test (ACT) result (well-controlled, not well-controlled, and very poorly controlled asthma). More details on that issue, including definitions of asthma severity and symptom control grading, are provided in the Supplementary Table S1.

The study was carried out while following the Declaration of Helsinki and the Ethics Committee of Jagiellonian University approved the protocol (approval number: KBET/151/B/2013). Furthermore, all subjects gave written informed consent to participate in the study.

### 2.2. Spirometry and Lung Computed Tomography (CT)

Spirometry and bronchial reversibility test (after 400 μg of albuterol) were assessed according to the standards of the American Thoracic Society [22], using a Jaeger MasterLab spirometer (Jaeger-Toennies GmbH, Hochberg, Germany). Persistent airflow limitation was defined as an FEV_1_/VC index below 0.7 or FEV_1_ less than 0.8 of the predicted value after the bronchodilator.

Lung computed tomography (CT) was performed after 400 μg albuterol administration, using 64-raw multidetector computed tomography (Aquilion TSX-101A, Toshiba Medical Systems Corporation, Otawara, Japan) in helical scanning mode (CT parameters: 64 × 0.5 mm collimation, the helical pitch of 53 and 0.5 s per rotation with standard radiation dose (150 ± 50 mAs and 120 kVp)). The automated AW Server program (Thoracic VCAR, General Electric Healthcare, Wauwatosa, WI, USA) was applied to quantify the cross-sectional geometry of the airways at the site of the right upper lobe apical segmental bronchus (RB1) and the right lower lobe basal posterior bronchus (RB10), including the lumen and wall area, average wall thickness, wall area ratio (WAR, i.e., average difference between the outer and inner areas divided by the outer area), and wall thickness ratio (WTR, i.e., wall thickness divided by the outer diameter) [23]. Spirometry and lung CT measurements were performed only in subjects with asthma.

### 2.3. Bronchofiberoscopy and Airway Sample Collection

Bronchofiberoscopy was also carried out in asthma patients only, according to the guidelines of the American Thoracic Society [24], using the bronchofiberoscope BF 1T180 (Olympus, Shinjuku, Japan) with local anesthesia (2% lidocaine) and with mild sedation (2.5–5 mg of midazolam, 0.05–0.1 mg of fentanyl i.v.). Bronchoalveolar lavage (BAL) was performed with 200 mL of 0.9% saline administered to the right middle lobe bronchus, and 2 or 3 endobronchial biopsies were taken from the right lower lobe (the carina between B9 and B10) during the procedure. The differential of BAL fluid cells was analyzed by using May–Grunwald–Giemsa-stained cytospin preparations (Thermo Scientific, Walthman, MA, USA; 1000 cells counted). The results were shown as a percentage of all inflammatory cells (except for epithelial cells). The BAL fluid supernatant was aliquoted and stored at −70 °C until analyzed.

Endobronchial biopsy specimens were formalin-fixed (Sigma-Aldrich, Saint Luis, MO, USA) and prepared for histological examination (e.g., hematoxylin-and-eosin staining) as previously described [23]. The microscope slides were photographed with a Nikon D5300 camera attached to the Zeiss Axioscope microscope with a 100× oil immersion lens. Images were analyzed by AnalySIS 3.2 software (Soft Imaging System GmbH, Muenster, Germany). The RBM thickness was measured along the airway epithelium layer, according to the orthogonal intercept method suggested by Ferrando et al. [25], using arbitrary distance units. For each patient, at least 30 individual RBM measurements were evaluated at intervals of 9.5 μm. The results were expressed as a harmonic mean, as defined in our previous publication [23].

### 2.4. Laboratory Investigations

#### 2.4.1. Basic Laboratory Tests

The complete blood cell count, the plasma concentration of fibrinogen, serum C-reactive protein (CRP), and immunoglobulin E (IgE) were measured in fasting blood samples by routine laboratory techniques. Serum and plasma samples were aliquoted and stored at −70 °C until analysis. Interleukin (IL)-4, IL-5, IL-6, IL-10, IL-12p70, IL-17A, and interferon (INF)-γ in serum and BAL samples were assessed by using commercially available high-sensitivity ELISA assays (eBiosciencea, Vienna, Austria). Periostin (a renowned marker of T2-immune response) was evaluated only in BAL by ELISA (Phoenix Pharmaceuticals, Burlingame, CA, USA). Similarly, citrullinated histone 3 (H3cit), a marker of neutrophil trap formation, was measured in serum, using an ELISA kit (Cayman Chemicals, Ann Arbor, MI, USA). Most BAL cytokine measurements were below the assay threshold (results are not shown in the tables).

#### 2.4.2. Coumarin Boronic Acid (CBA) Assay

To assess the oxidative potential of proteins in serum, we applied the real-time monitoring of HP formation, using the CBA-based assay, as previously described [21,26]. Briefly, 50 μL of serum sample was transferred to 96-microwell black plates and mixed with 150 μL phosphate buffer (50 mM, pH 7.4) containing catalase (100 units/mL), DTPA (diethylenetriaminepentaacetic acid; 0.1 mM), and non-fluorescent CBA as a substrate (0.8 mM). The fluorescence intensity of the reaction product, that is, the 4-hyroxycoumarin (COH), was measured (Ex/Em: 360 nm/465 nm) at 10 min intervals, using a plate reader (ClarioStar, BMG Labtech, Ortenberg, Germany), for 20 h. Each serum sample was analyzed in duplicates, and the arithmetic means presented the experiment’s outcome. In each case, the proper background fluorescence was subtracted, and the sample fluorescence was adjusted to the total protein concentration in the serum.

The intensity of fluorescent product generation demonstrated exponential growth that was fitted to the logistic growth model. The resulting sigmoidal logistic growth curve and growth dynamics were described by the three parameters: (1) saturation level, ‘K concentration’, representing a numerical upper limit of growth; (2) rate factor, ‘R’, which describes the velocity of the fluorescent product growth; and (3) the area under the curve until saturation level, representing a cumulative generation of the CBA fluorescent product over time.

### 2.5. Statistical Analysis

Statistical analysis was performed with Statistica TIBCO 13.3 software (TIBCO Software Inc., Palo Alto, CA, USA) and R (version 3.6.1). We used the Shapiro–Wilk test to verify the distribution of the data. As appropriate, continuous variables were reported as a median with 0.25–0.75 quartiles or as a mean with standard deviation. They were compared by using the Mann–Whitney U test or unpaired t-test, respectively. Categorical variables were given as percentages and compared by χ^2^ test. The variables of the CBA assay were Box-Cox transformed, and one-way covariance analysis (ANCOVA) was performed to adjust for potential confounders, including age, sex, and BMI. To evaluate the relationship between continuous variables, a Spearman rank correlation test or Pearson correlation tests were performed as applied. The cutoff points for the oxidative stress parameters were calculated based on the receiver operating characteristic (ROC) curve to estimate the odds ratio (OR) with a 95% confidence interval (CI). Independent determinants of cumulative in time concentration, K concentration, and rate factor R were established in multivariate linear regression models, built using a stepwise forward selection procedure, verified by Snedecor’s F-distribution; R^2^ was evaluated as a measure of variance. Unconditional multivariate logistic regression model and one-way variance analysis (ANOVA) were used to analyze the independent impact of comorbidities, including hypertension, diabetes mellitus, hypercholesterolemia, and oral steroid and statin therapy on evaluating oxidative stress, respectively. Results that had a *p*-value less than 0.05 were considered statistically significant.

## 3. Results

### 3.1. Study Participants

Asthma patients and control subjects were well matched according to the demographic variables, including age, sex, body mass index (BMI), and the area of residence, i.e., rural vs. urban area (Table 1). Likewise, the prevalence of other chronic comorbidities (such as arterial hypertension or diabetes mellitus) was similar. Finally, all study participants were current nonsmokers (>5 years).

### 3.2. Clinical Characteristics and Airway Remodeling in Asthma Patients

Among the 74 asthma patients included, 29 (39%) had severe disease (Table 2), and 37 (50%) had persistent airflow limitation. The median duration of asthma was 10 years, and about half of the patients were atopic. Only one-third of the subjects enrolled evaluated their asthma as well-controlled based on the asthma control test, while one-fourth had a very poorly controlled disease.

Based on the BAL cell differential count data (Table 2), 26% of asthma patients showed eosinophilic inflammation (that is, ≥2% of eosinophils), including 15% with neutrophil admixture (mixed inflammation); 28% had a pure neutrophilic (≥4% neutrophils), and 46% had a pauci-granulocytic variant.

In the Supplementary Materials, we provided the clinical characteristics of asthma patients with the division into mild, moderate, and severe disease staging. 

**Figure 1:**
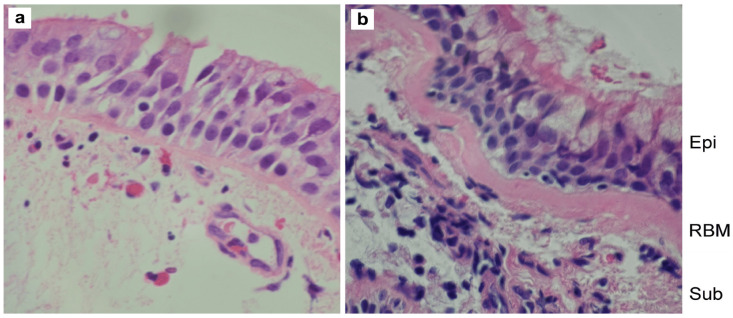
Representative pictures of endobronchial biopsy specimens in a control subject (**a**) and asthma patient (**b**); the reticular basement membrane (RBM) is thicker in asthma. Other abbreviations: Epi—epithelium, Sub—subepithelium.

Table 2 also summarizes essential measures of structural airway remodeling, as evidenced by CT imaging and airway biopsy specimens. Unfortunately, in this study, we did not collect those data in the control group. However, compared to control datasets in our previous report [27], which was conducted by using a similar methodology, CT imaging parameters suggest mild-to-moderate changes in airway geometry (10% difference on average, *p* < 0.05), whereas RBM thickness is ~30% thicker in asthmatics compared to controls (*p* < 0.001). In Figure 1, we depict representative pictures of RBM measures in control and asthma individuals. 

As expected, the referenced airway CT measures in RB1 and RB10 correlated well with each other (e.g., WTR: r = 0.4, *p* < 0.001) and with spirometry values (e.g., FEV_1_: r = −0.34, *p* = 0.007 and r = −0.3, *p* = 0.01, for WTR of RB1 and RB10, respectively). 

At the same time, neither CT nor spirometry values were linked with RBM thickness. 

### 3.3. Increased Systemic Generation of Protein Hydroperoxides in Asthma

**Figure 2:**
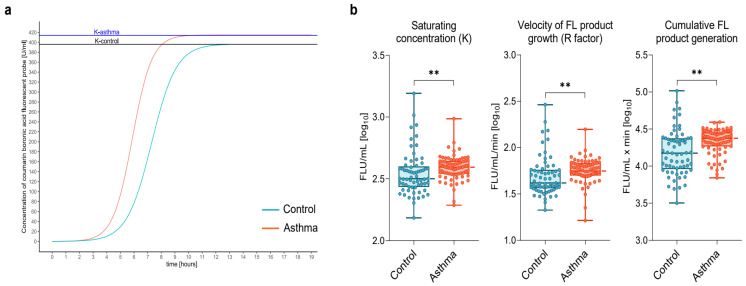
(**a**) Representative curve of fluorescent product generation in the real-time coumarin boronic acid (CBA) assay of asthma patient and control individual (K—saturating concentration). (**b**) Saturating concentration (K), growth velocity (R factor), and cumulative fluorescent (FL) product generation in the real-time coumarin boronic acid (CBA) assay in asthma and control subjects. Abbreviations: FL—fluorescent, FLU—fluorescent unit, K—saturating concentration, R—velocity of fluorescent product growth, ** *p* < 0.001.

The fluorescent intensity of the CBA-assay product, reflecting HP generation, increased quickly both in the asthmatics and controls (Figure 2a). However, the velocity of fluorescence growth, reflected by the factor R, was 35% higher, and the saturating concentration (K) increased by 23% more in asthmatics than in controls (*p* < 0.001, using the Mann–Whitney U test; *p* = 0.01 and *p* = 0.009, respectively, using ANCOVA after adjustment for age, sex, and BMI) (Figure 2b). 

Compared to the control subjects, asthma patients showed an odds ratio (OR) of 3.14 (95%CI: 1.98–4.97; *p* < 0.001) for having a higher R factor, defined as values above the cut-off point of 42.4 fluorescence (FL) unit (U)/mL/min. Similarly, asthma patients had an OR of 2.57 (95%CI: 1.7–3.88; *p* < 0.001) for having increased K concentration, using a cutoff point of 322 FLU/mL. 

Furthermore, we demonstrated a 58% higher cumulative in time HP generation (area under the curve) (Figure 2b, *p* < 0.001, using Mann–Whitney U test; *p* = 0.01, using ANCOVA after adjustment for age, sex, and BMI). 

**Figure 3:**
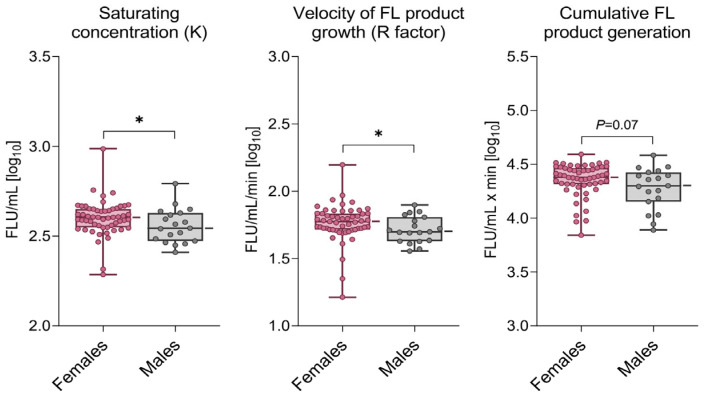
Hydroperoxides’ generation in the coumarin boronic acid (CBA) assay was higher in females than males in the asthma group; * *p* < 0.05; for other abbreviations, see legend in Figure 1.

In the asthma group, the dynamics of HP generation were ~20% higher, and K concentration was increased by ~15% in females (Figure 3). 

Other demographic variables and comorbidities had no impact on the CBA assay results.

### 3.4. Systemic Protein Hydroperoxides Generation Was Related to Bronchial Obstruction, Airway Geometry, and Bronchoalveolar Lavage Fluid Biomarkers

Next, we analyzed associations of systemic oxidative stress measures with clinical characteristics of asthma, lung function, airway remodeling indices, and BAL biomarkers.

**Figure 4:**
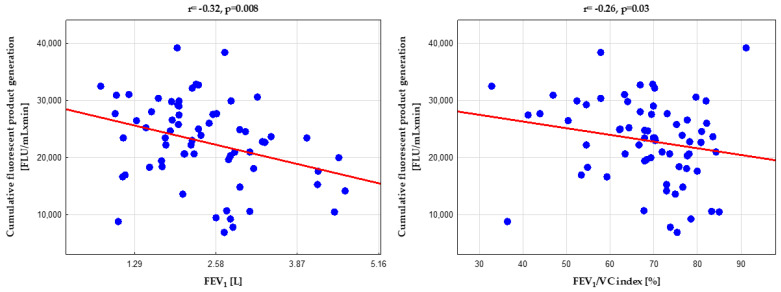
Inverse correlation between hydroperoxide generation in coumarin boronic acid (CBA) assay and lung function; for abbreviations, see the legend in Figure 2 and Table 2.

Surprisingly, the dynamics of HP generation were not related to the severity of asthma, symptom control, or asthma medications used. However, we detected a weak inverse correlation with airway obstruction spirometry indices (Figure 4). 

Furthermore, asthma patients with persistent airflow limitation showed a 19% increase in cumulative HP formation (*p* = 0.02).

Oxidative stress measures were not associated with RBM thickness or collagen I deposit in bronchial biopsy specimens. They were also not directly associated with lung CT parameters. However, multiple regression models showed that WAR or WTR in RB1 could be an independent determinant of higher HP formation dynamics, as presented in Table 3 (a model for the R factor).

**Figure 5:**
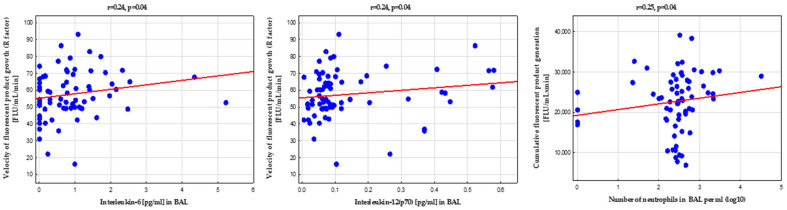
Positive correlations between dynamics in hydroperoxide generation in the real-time coumarin boronic acid (CBA) assay and bronchoalveolar lavage fluid biomarkers (interleukin [IL]-6, IL-12, and BAL neutrophilia); for abbreviations, see the legend in Figure 2 and Table 2.

Among BAL biomarkers, the HP generation was weakly positively associated with the IL-6 and IL-12(p70) concentrations and neutrophil count (Figure 5). Additionally, BAL IL-6 appears to be an independent determinant of higher HP formation, as shown in Table 3.

### 3.5. Complex Regulation of Circulating Protein Hydroperoxides Generation by Peripheral Blood Biomarkers

Then we investigated the relation of HP generation in asthma to laboratory variables measured in the systemic circulation, including blood cell counts, inflammatory biomarkers, and atherosclerosis risk factors, such as glucose level and lipid profile. 

As expected, asthma patients were characterized by increased blood eosinophilia and serum IgE compared to the controls (Table 4). Interestingly, the serum CRP concentration was also marginally elevated in the patients (*p* = 0.04). On the other hand, glucose and total cholesterol levels and blood cytokines were comparable in both study groups, except for IL-10, which was higher in asthmatics. Similarly, the concentration of H3cit was increased in patients, suggesting neutrophil activation and the formation of extracellular traps [28].

**Figure 6:**
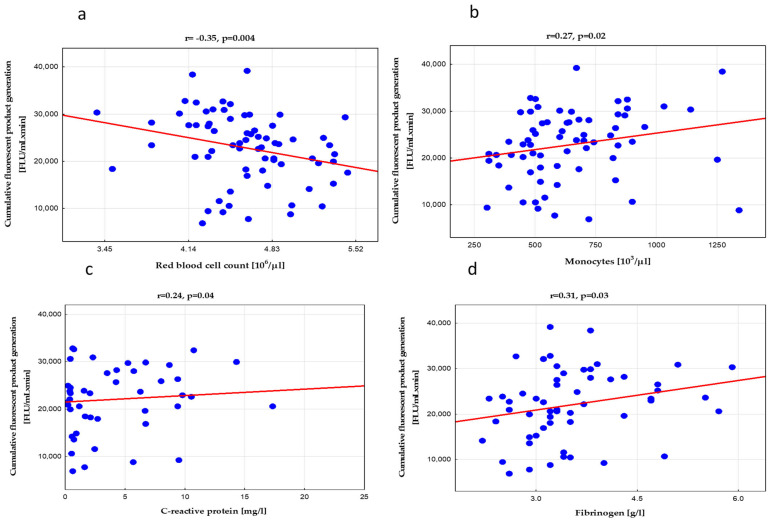
Relationships between cumulative hydroperoxide generation in the real-time coumarin boronic acid (CBA) assay and red blood cell count (**a**) peripheral blood monocyte count (**b**), circulating C-reactive protein (**c**), and fibrinogen (**d**); for abbreviations, see the legend in Figure 2 and Table 2.

Interestingly, systemic HP generation in asthma showed a clear negative association with the red blood cell (RBC) count (Figure 6a) and hemoglobin level. This relationship applied to all CBA assay parameters and was not seen in the control grroup. 

Furthermore, we demonstrated a weak positive association between HP formation dynamics and blood monocyte count (Figure 6b) and some nonspecific markers of inflammation, such as serum CRP (Figure 6c) or plasma fibrinogen (Figure 6d). In contrast, it was not directly related to renowned T2 immune response measures in blood, such as eosinophilia.

In Table 5, we demonstrate the independent determinants among the measured blood biomarkers of increased susceptibility to HP formation (R factor) in a multiple regression model. Interestingly, various variables predicted an increase in the dynamics of systemic HP generation, including blood monocyte count, H3cit, and IL-17A, as well as total cholesterol levels. At the same time, the RBC count and, to a much lesser extent, the blood eosinophil count inversely impacted the R factor. 

Eventually, we performed a combined analysis by using a multivariable stepwise regression model, considering both the airway and systemic measures investigated in the study that could impact HP generation. As presented in Table 6, susceptibility to increased HP formation was determined best by lower spirometry values (e.g., FEV_1_), elevated IL-6 in BAL, and higher circulating H3cit. As expected, the RBC count had a substantial negative contribution to that analysis.

## 4. Discussion

The present study demonstrates an increase in the generation of amino acid HP in the peripheral blood of asthmatic patients, reflecting an enhanced systemic oxidative stress response in that disease. In asthma, increased ROS formation by inflammatory cells has already been described in both the airways and systemic circulation [5,13,18]. Here, we show that it can be assessed reliably in the peripheral blood by using a recently developed technically undemanding real-time CBA assay [21,29,30]. Furthermore, we have investigated whether elevated oxidative stress in asthma, related to increased HP generation, is determined by clinical manifestations of the disease, inflammatory patterns, and various measures of airway remodeling. 

In our study, increased systemic oxidative stress was associated with different variables related to asthma, including bronchial obstruction, airway geometry, and unspecific inflammatory biomarkers analyzed in the lungs and blood. Nevertheless, it was not associated with asthma severity score and symptom control, suggesting that higher blood oxidative stress in asthma is a feature of that disease per se. That is an unexpected finding since asthma is an inflammatory disease of the airways. However, many reports, including our previous research, have indicated that asthma is associated with higher inflammatory biomarkers in circulation [31]. In addition, this low-grade systemic response was related to the prothrombotic state [32] that was documented previously in that disease, as well as in the epidemiological studies [15,16,17]. Thus, local airway inflammation is likely associated with a variable degree of systemic response in asthma, activating blood leukocytes further and leading to higher HP production. 

On the other hand, lower FEV_1_/VC values, reflecting more advanced airway obstruction and thicker bronchial walls in RB1, were independent determinants of higher susceptibility to HP generation. This observation suggests that objective indicators of more severe disease forms may be related, indeed, to the higher circulating oxidative stress capacity. At the same time, various laboratory variables in peripheral blood and BAL predicted higher HP formation. Among them, the most important were those related to innate immunity and unspecific inflammation, such as BAL neutrophilia and blood monocyte count, as well as circulating CRP, fibrinogen, IL-17A, and H3cit. The latter is a novel biomarker of neutrophil activation and extracellular trap formation [28]. The relation of circulating oxidative stress to CRP in asthmatics is not surprising. A similar association was previously shown in other conditions, e.g., in healthy heavy workers [33]. Conversely, in controls, we did not record such a relationship. However, the six control subjects who had HP generation very high, above the fourth-quartile cutoff point in the asthmatics (Figure 2b), were characterized by the highest CRP values (all within the normal range), as compared to the remaining control subjects (2.65 [1.4–4.6] vs. 1 [1–1.7] mL/L, *p* = 0.006). Interestingly, those six controls have not reported any chronic inflammatory or acute disease or significant clinical symptoms and did not differ in the prevalence of internal comorbidities or other laboratory variables, except for CRP. Therefore, they were not excluded from the control group.

Interestingly, in our study, a higher oxidative stress response in asthma was determined by an increased total cholesterol level in serum. Since hypercholesterolemia and oxidative stress are well-known factors leading to endothelial dysfunction and atherosclerosis [9,14], we speculate that elevated HP generation in asthma might unfavorably affect the cardiovascular system. However, advanced observational and experimental studies are needed to verify this hypothesis. 

On the other hand, the negative association of HP generation with blood eosinophilia in a multiple regression model suggests that oxidative stress in asthma is not related to the T2 response [1,12]. Thus, our report is consistent with several epidemiological studies that indicate that an increased risk of cardiovascular diseases in asthma occurs primarily in the late-onset asthma phenotype [34,35] or even in women with adult asthma [17], more frequently representing the non-T2 phenotype [36,37].

The lack of a relationship between HP formation and the histological characteristics of airway remodeling is another surprising finding that deserves a comment. Protein and lipid oxidation were previously linked to pro-inflammatory airway epithelial and endothelial cell modification [5]. Therefore, increased oxidative stress could lead to airway structural changes in the airways, such as the thickening of the RBM. However, the characteristics and role of structural alterations of the bronchial wall in asthma remain unknown. Previously, we have shown that RBM thickening did not depend on the asthma duration or lung function [12]. The current study likewise documents that it is also not linked to asthma severity. Notably, our reports align with former data, such as those published by Payne et al. [38]. They demonstrated that RBM thickening was present even in young children with asthma to a similar extent as seen in milder adult asthmatics and independently of asthma duration, severity, and lung function [38]. Therefore, it seems that changes in RBM occur early during the disease, e.g., as a response to ongoing airway inflammation, and do not progress further, due to the implemented anti-inflammatory treatment. Therefore, airway inflammation may promote ROS overproduction during a stable course of asthma but not further RBM thickening. 

One of the strongest associations in our study is a clear inverse relationship between the potential for oxidative stress and RBC count. It indicates a substantial contribution of the antioxidant system of RBCs in balancing enhanced ROS generation. In the systemic circulation, the RBCs are continuously exposed to exogenous and endogenous sources of ROS, e.g., released from activated locally inflammatory cells. However, they possess an extensive antioxidant system, involving nonenzymatic antioxidants, for example, glutathione and enzymes, such as superoxide dismutase, catalase, glutathione peroxidase, and peroxiredoxin-2 [39]. Our data suggest that the antioxidative potential of RBC could diminish the lung-originated oxidative stress in asthma by counteracting ROS, at least to some extent. Therefore, it might be particularly insufficient in asthma patients with anemia. 

Whether enhanced oxidative stress in asthma requires any therapeutic modifications is still unknown. However, some interventions have been suggested, including dietary changes, antioxidant vitamins, other antioxidant drugs and supplements, and even radon exposure, with varying results [40]. For example, nutritional studies suggest that asthmatic children with low dietary intake of vitamins C and E and other antioxidants have worse asthma symptoms; however, therapy with these vitamins was ineffective [40]. Another large epidemiological study prospectively documented the lower prevalence of asthma in those with higher α-tocopherol and Lp-PLA_2_ activity in peripheral blood at baseline [10]. At the same time, two longitudinal studies of dietary intake demonstrated inconsistent results with asthma risk. The Nurses’ Health Study showed a higher asthma rate in those with a lower dietary intake of vitamin E [41], while in the E3N study, no such association was documented [42].

Finally, GINA [1] does not recommend any antioxidant supplements to decrease asthma-induced oxidative stress. Therefore, proper disease control, dietary intake of natural antioxidants in fruits and vegetables, and minimizing exposure to environmental pollution and tobacco smoke remain paramount.

### Study Limitation

The main limitation of this study is the analysis focused only on HP products in proteins but not lipids or nucleic acids. Furthermore, we did not analyze endogenous or exogenous antioxidants, including genetic polymorphisms [5,10]. However, our study, a pilot in nature, relied on complex analysis, estimating the total harmful effects of ROS on proteins and considering the impacts of all available pro- and antioxidant factors in circulating blood. Furthermore, the CBA assay was measured once; therefore, we cannot assess its variability over time, depending on the course of asthma. Finally, we did not analyze the airway parameters in controls, obviously due to ethical reasons. However, since asthma is a chronic inflammatory lung disease, we believe that our research is valuable and worth publishing. Those factors limit the depth of the investigation but not the study’s main findings.

## 5. Conclusions

We have shown increased levels of amino acid and protein hydroperoxides measured in the serum of asthmatic patients, using a recently developed technically undemanding real-time CBA assay. Furthermore, estimated oxidative stress was related to BAL and blood inflammatory biomarkers, spirometry values, and CT airway geometry measures, but not asthma severity or histological indices of airway remodeling. More prospective and experimental studies are needed to verify the biological role of increased circulating oxidative stress in the clinical course of asthma and extrapulmonary complications.

## Figures and Tables

**Table 1:** Demographic characteristics and comorbidities in asthmatic patients and control individuals.

Variables	Patients *n* = 74	Controls *n* = 65	*p*-Value
Age, years	53.5 ± 13.1	51.4 ± 12.3	0.63
Male gender, *n*(%)	19 (26)	23 (35)	0.22
Body mass index, kg/m^2^	26.9 ± 4.6	25.9 ± 3.4	0.1
Past smoking, *n*(%)	13 (18)	15 (23)	0.3
Pack-years of smoking, *n*	0 (0–0)	0 (0–0)	0.59
Living primarily in inner-city environments, *n*(%)	39 (53)	38 (58)	0.57
Internal medicine comorbidities			
Hypertension, *n*(%)	34 (46)	25 (38)	0.67
Diabetes mellitus, *n*(%)	11 (15)	8 (12)	0.68
Hypercholesterolemia, *n*(%)	21 (28)	15 (23)	0.52

Categorical variables are presented as *n*-numbers (percentages); continuous variables are presented as median and interquartile range, or mean and standard deviation, as appropriate.

**Table 2:** Clinical characteristics of asthmatic patients, including airway imaging and histo(cyto)logy.

Asthma duration, years	10 (5–20)
Atopy, *n*(%)	39 (53%)
Severe asthma, *n*(%)	29 (39%)
Asthma severity (GINA)	
Mild, *n*(%)	18 (24%)
Moderate, *n*(%)	27 (36%)
Severe, *n*(%)	29 (39%)
Asthma symptom control ^§^	
Well-controlled asthma, *n*(%)	22 (30%)
Not-well controlled asthma, *n*(%)	33 (44%)
Very-poorly controlled asthma, *n*(%)	19 (26%)
Spirometry values	
FEV_1_ before bronchodilator, % of the predicted value	81.8 (66.6–99.4)
FEV_1_ after bronchodilator, % of the predicted value	91.9 (73.2–104.1)
VC before bronchodilator, L	3.2 (2.6–3.97)
VC after bronchodilator, L	3.36 (2.7–4)
FEV_1_/VC (before bronchodilator)	65.8 (57.4–72.6)
FEV_1/_VC (after bronchodilator)	69.9 (63.2–77.5)
Computed tomography airway remodeling indices	
*The right upper lobe apical segmental bronchus (RB1)*	
Lumen area, mm^2^	12.5 (10–16)
Wall area, mm^2^	34.8 (27.9–45.4)
Wall thickness, mm	1.9 (1.7–2.1)
Wall thickness ratio (WTR)	24.1 ± 2.7
Wall area ratio (WAR)	73.2 ± 5.6
*The right lower lobe basal posterior bronchus (RB10)*	
Lumen area, mm^2^	12.5 (9–18)
Wall area, mm^2^	35.1 ± 12.3
Wall thickness, mm	1.8 (1.6–2.1)
Wall thickness ratio (WTR)	23.6 (22–25.9)
Wall area ratio (WAR)	72.3 (68.5–76.9)
Bronchial biopsy histology	
Reticular basement membrane (RBM) thickness, μm ^¥^	6.49 (5.3–7.86)
Collagen I staining, % of the stroma showing reactivity	30 (20–60)
Bronchoalveolar lavage fluid (BAL) cellularity ^#^	
Macrophages, %	85 (72–93)
Lymphocytes, %	8 (4–15)
Neutrophils, %	3 (2–5)
Eosinophils, %	1 (0.1–2)
Eosinophils ≥2% in BAL, *n*(%)	17 (37.5%)
Neutrophils ≥4% in BAL, *n*(%)	29 (43.3%)
Bronchoalveolar lavage fluid biomarkers ^†^	
Periostin, ng/mL	0.85 (0.75–0.97)
Interleukin-6, pg/mL	0.75 (0.1–1.19)
Interleukin-12(p70), pg/mL	0.078 (0.05–0.12)
Asthma therapy	
Oral corticosteroids	14 (19%)
Inhaled corticosteroids (persistent use)	68 (92%)
Long-acting β_2_-agonists (persistent use)	54 (73%)
Antileukotrienes	10 (14%)
Theophylline	8 (11%)
Long-acting anticholinergics (persistent use)	5 (7%)

Categorical variables are presented as numbers (percentages); continuous variables are presented as median and 0.25–0.75 quartiles, or mean and standard deviation, as appropriate. Abbreviations and references: BAL—bronchoalveolar lavage fluid, FEV_1_—forced expiratory volume in one second, GINA—Global Initiative for Asthma, L—liter, VC—vital capacity; ^§^ asthma symptom control (assessed based on Asthma Control Test results); ^#^ BAL cell differential data available in 67 asthma subjects; ^†^ BAL fluid levels of interleukin (IL)-4, IL-5, IL-10, and IL-17A and interferon γ were below the detection threshold (data not shown); ^¥^ RBM available in 45 asthma subjects.

**Table 3:** Multiple linear regression model for a relative increase of fluorescent product growth velocity (R factor) in the real-time CBA assay in asthma patients. Presented variables are documented as independent determinants; however, they explain only 16% of the R factor variability.

Fluorescent Product Growth Velocity (R Factor)		
	β (95% CI)	R^2^
FEV_1_, %	−0.18 (−0.31 to −0.04)	0.16
Wall thickness ratio (WTR), RB1	0.22 (0.08 to 0.36)	
Interleukin 6, BAL, pg/mL	0.28 (0.14 to 0.41)	
Adjustment statistics	F = 2.9, *p* < 0.001	

The resulting standardized regression coefficient (β) with 95% confidence interval (95% CI) for a factor (independent variable) indicates the increase/decrease in standard deviations (SDs) of a dependent variable (R factor) when that particular factor increases by 1 SD and all other variables in the model remain unchanged. Abbreviations: RB1—the right upper lobe apical segmental bronchus; for other abbreviations, see footnote to Table 2.

**Table 4:** Laboratory parameters in asthmatic patients and control subjects.

	Patients *n* = 74	Controls *n* = 65	*p*-Value
Basic laboratory tests			
Hemoglobin, g/dL	13.5 (13–14.4)	13.9 ± 1.28	0.09
Red blood cell count, 10^6^/μL	4.61 ± 0.43	4.6 ± 0.38	0.83
White blood cell count, 10^3^/μL	6.68 (5.6–7.96)	5.48 (4.82–6.64)	<0.001 **
Eosinophils, 10^3^/μL	275 (135–470)	15 (9–80)	<0.001 **
Monocytes, 10^3^/μL	600 (485–815)	480 (430–670)	<0.001 **
Blood platelets, 10^3^/μL	218 (191–247)	232 (203–293)	0.01 *
C-reactive protein, mg/L	2.55 (0.58–8.67)	1 (0.9–1.7)	0.04 *
Immunoglobulin E, IU/mL	71.5 (29.4–380)	22.6 (18.5–53.5)	0.001 *
Glucose, mmol/L	5 (4.65–5.55)	5.17 ± 0.54	0.63
Total cholesterol, mmol/L	4.83 ± 0.98	4.8 (4.25–5.35)	0.74
Low-density lipoprotein cholesterol, mmol/L	2.61 ± 0.76	3.38 ± 0.96	<0.001 **
High-density lipoprotein cholesterol, mmol/L	1.34 (1.09–1.61)	1.58 ± 0.41	0.01 *
Triglycerides, mmol/L	1.4 (1–2)	1.09 (0.82–1.42)	<0.001 **
Biomarkers in serum ^§^			
Interleukin-6, pg/mL	0.78 (0.45–2.09)	0.73 ± 0.52	0.31
Interleukin-10, pg/mL	0.57 (0.25–0.97)	0.005 (0.005–0.01)	<0.001 **
Interleukin-12(p70), pg/mL	0.005 (0.005–1.3)	0.005 (0.005–1.69)	0.75
Interleukin-17A, pg/mL	0.005 (0.005–0.12)	0.005 (0.005–0.06)	0.87
Interferon-γ, pg/mL	0.005 (0.005–0.28)	0.11 (0.005–0.27)	0.66
Citrullinated histone H3, ng/mL	16.3 (10.7–19.8)	12.8 (8.2–17.6)	0.04 *

Variables are presented as median and interquartile range, or mean and standard deviation, as appropriate. References: ^§^ all serum measurements of interleukin (IL)-4 and IL-5 were below the assay threshold (data not shown); ** *p* < 0.001; * *p* < 0.05 and *p* ≥ 0.001.

**Table 5:** Multiple linear regression model for a relative increase of fluorescent product growth velocity (R factor) in the real-time CBA assay in asthma patients. Presented variables were documented as independent determinants; they explain 33% of R factor variability.

Fluorescent Product Growth Velocity (R Factor)		
	β (95% CI)	R^2^
Monocyte count, 10^3^/μL	0.14 (0.02 to 0.28)	0.33
Red blood cell count, 10^6^/μL	−0.58 (−0.73 to −0.43)	
Blood eosinophilia, 10^3^/μL	−0.23 (−0.41 to −0.06)	
Citrulinated histone 3, ng/mL	0.22 (0.07 to 0.37)	
Interleukin 17A, pg/mL	0.26 (0.13 to 0.4)	
Total cholesterol, mmol/L	0.22 (0.08 to 0.37)	
Adjustment statistics	F = 3.3, *p* = 0.01	

For data interpretation, see footnote to Table 3.

**Table 6:** Multiple linear regression model for a relative increase of fluorescent product growth velocity (R factor) in the real-time CBA assay in asthma patients, considering all study variables.

Fluorescent Product Growth Velocity (R Factor)		
	β (95% CI)	R^2^
FEV_1_, %	−0.21 (−0.34 to −0.07)	0.26
Red blood cell count, 10^6^/μL	−0.26 (−0.39 to −0.12)	
Citrulinated histone 3, ng/mL	0.35 (0.23 to 0.47)	
Interleukin 6, BAL, pg/mL	0.19 (0.08 to 0.31)	
Adjustment statistics	F = 5.04, *p* = 0.002	

For data interpretation, see footnote to Table 3. For the abbreviations, see footnote to Table 2.

## Data Availability

The data presented in this study are available upon request from the corresponding author. The data are not publicly available due to patients’ origin.

## Supplementary Materials

The following supporting information can be downloaded at https://www.mdpi.com/article/10.3390/biomedicines10071499/s1. Table S1: Clinical characteristics of asthmatic patients divided into mild, moderate, and severe disease stages, including airway imaging and histo(cyto)logy.
