# Supplementary material for: Increased Oxidative Stress in Asthma—Relation to Inflammatory Blood and Lung Biomarkers and Airway Remodeling Indices
Source: Biomedicines. 2022 Jun 24;10(7):1499. doi: 10.3390/biomedicines10071499 (PMC9312921; doi:10.3390/biomedicines10071499)
Supplement: Supplementary file 1 [file biomedicines-10-01499-s001.zip › biomedicines-1669798-supplementary.pdf]

biomedicines

Increased oxidative stress in asthma – relation to inflammatory blood and lung biomarkers and airway remodeling indices

Stanisława Bazan-Socha[1*#§], Krzysztof Wójcik[1#§], Magdalena Olchawa[2], Tadeusz Sarna[2], Jakub Pięta[3], Bogdan Jakieła[1#], Jerzy Soja[1&], Krzysztof Okoń[4], Jacek Zarychta[1#,5], Lech Zaręba[6], Michał Stojak[7], Daniel P. Potaczek[8], Jan G. Bazan[6], Magdalena Celińska-Lowenhoff[1#],

Supplementary file

Materials and Methods

Study participants

Diagnosis of asthma and disease severity were established based on the current Global Initiative for Asthma (GINA) guideline [1]. "Mild" asthma was defined as a mild disease treated with a low daily dose of inhaled corticosteroids (ICS) (<250 μg of fluticasone propionate [FP] [dry powder inhaler] or equivalent) with a short-acting β2-agonist as a rescue medication or combination of ICS/formoterol used on demand. "Moderate" asthma was defined as a mild persistent disease treated constantly with a low (combined with long-acting β2-agonists) or medium dose of ICS (250-500 μg of FP or equivalent). "Severe" asthma was defined as asthma that is uncontrolled despite adherence with an optimized high daily dose of ICS (>500 μg of fluticasone propionate or equivalent) with using of long-acting β2-agonist or that worsened when high dose ICS treatment is decreased.

Asthma symptom control was assessed based on the Asthma Control Test (ACT) result. Scores 20-25 were classified as "well-controlled asthma", 16-19 as "not well-controlled", while 5-15 as "very-poorly controlled asthma".

Diabetes mellitus was defined as the current use of insulin, oral hypoglycemic medications, or fasting serum glucose >7.0 mmol/l. Arterial hypertension was defined based on a history of hypertension (blood pressure >140/90 mmHg) or administration of antihypertensive treatment. Hypercholesterolemia was defined as previously diagnosed and treated with statins or serum total cholesterol >5.2 mmol/l. Liver injury was diagnosed if serum alanine aminotransferase was elevated (at least two times above the upper limit of the reference range). Finally, coronary heart disease was defined as a documented history of coronary stenosis or stable angina.

Results

Patients

In Table S1, we have shown clinical asthma-related variables in 74 patients, divided into mild, moderate, and severe disease stages.

As expected, the severe disease, compared to mild asthma, was characterized by a higher prevalence of a very-poorly controlled disease, despite 48% of those patients being treated with oral steroids. Furthermore, eosinophilic asthma was diagnosed more frequently in the severe disease form.

On the contrary, the asthma subgroups did not differ in disease duration (median ten years) and airway remodeling indices measured in lung CT or histology (Table S1).

Table S1. Clinical characteristics of asthmatic patients divided into mild, moderate, and severe disease stages, including airway imaging and histo(cyto)logy.

	**Asthma severity (GINA)**			**p-value**		
	Mild n=18 (24%)	Moderate n=27 (36%)	Severe n=29 (39%)	Mild vs. Moderate	Mild vs. Severe	Moderate vs. Severe
Asthma duration, years	10 (5-13)	8 (5-22)	10 (7-25)	0.97	0.15	0.82
Atopy, n(%)	10 (56%)	17 (63%)	12 (41%)	0.19	0.27	0.1
**Asthma symptom control §**						
Well-controlled asthma, n(%)	9 (50%)	10 (37%)	5 (17%)	0.19	**0.008***	0.05
Not-well controlled asthma, n(%)	8 (44%)	11 (41%)	13 (45%)	0.42	0.47	0.38
Very-poorly controlled asthma, n(%)	1 (6%)	6 (22%)	11 (38%)	0.07	**0.007***	0.1
**Spirometry values**						
FEV_1 before bronchodilator, % of the predicted value	96.4 (73.9-113)	80.6 (64.6-99.5)	74.2 (56.7-85.2)	0.3	**0.03***	0.93
FEV_1 after bronchodilator, % of the predicted value	104.4 (90.8-110.2)	86.1 (72.3-104.7)	86.3 (71.2-96.5)	0.08	**0.02***	0.99
VC before bronchodilator, L	3.81 (2.7-4.2)	3.11 (2.7-4.1)	3.12 (2.6-3.4)	0.99	0.21	0.66
VC after bronchodilator, L	3.76 (2.7-4.7)	3.3 (2.6-4.1)	3.14 (2.7 -3.6)	0.77	0.13	0.88
FEV_1/VC (before bronchodilator)	71.3 (63.5-75.5)	66.7 (54.4-75.6)	62.3 (52-70.5)	0.57	0.1	0.99
FEV_1/VC (after bronchodilator)	77.5 (67.8-80.9)	69.9 (57.7-76.4)	68 (60-73.3)	0.1	**0.03***	0.99
**Computed tomography airway remodeling indices**						
The right upper lobe apical segmental bronchus (RB1)						
Lumen area, mm^2	11.45 (10-15.55)	12.2 (10.5-15.15)	13 (9.4-18.8)	0.99	0.95	0.99
Wall area, mm^2	34.8 (27.35-38.85)	33.45 (30.1-45.9)	38 (27.2-45.6)	0.99	0.93	0.85
Wall thickness, mm	1.9 (1.6-2)	1.9 (1.7-2.2)	1.8 (1.7-2.2)	0.99	0.99	0.99
Wall thickness ratio (WTR)	22.8 (21.9-24.7)	24.6 (22.25-26.65)	24.1 (23.1-25.7)	0.19	0.27	0.99
Wall area ratio (WAR)	70.6 (69-75.2)	74.4 (69.75-78.35)	73.15 (71.3-76.5)	0.36	0.62	0.99
The right lower lobe basal posterior bronchus (RB10)						
Lumen area, mm^2	12.1 (9.3-14.4)	13.1 (9.1-17)	11.25 (8.3-20.6)	0.53	0.58	0.52
Wall area, mm^2	32.5 (22.25-38.5)	35.7 (27-42.1)	36.15 (25.8-47.4)	0.83	0.70	0.48
Wall thickness, mm	1.75 (1.5-1.9)	1.8 (1.6-2.1)	1.8 (1.6-2.2)	0.99	0.99	0.99
Wall thickness ratio (WTR)	23.95 (22.95-25.5)	23.2 (21.2-26)	23.5 (22-25.6)	0.99	0.99	0.99
Wall area ratio (WAR)	72.85 (70.65-76.15)	71.2 (66.6-77.1)	72.15 (68.5-76.2)	0.99	0.99	0.99
**Bronchial biopsy histology**						
Reticular basement membrane (RBM) thickness, mm¥	6.82 (5.8-8.3)	6.09 (5.1-7.6)	6.49 (5.3-7.86)	0.9	0.46	0.63
Collagen I staining, % of the stroma showing re-activity	40 (20-75)	30 (20-60)	40 (20-60)	0.5	0.99	0.29
**Bronchoalveolar lavage fluid (BAL) cellularity#**						
Macrophages, %	90 (74-94)	85.5 (77-93)	82 (52.3-89)	0.78	0.06	0.55
Lymphocytes, %	7 (4-16.5)	8.5 (4-15.5)	8.25 (4.75-12.5)	0.99	0.99	0.99
Neutrophils, %	2 (1-4)	3 (2-4.5)	4.25 (2.5-12)	0.06	0.07	0.32
Eosinophils, %	0.3 (0.1-1)	0.5 (0-1)	1.25 (0.5-5.75)	0.06	**0.02***	0.08
Eosinophils ≥2% in BAL, n(%)	2 (11.1%)	4 (14.8%)	11 (37.9%)	0.38	**0.02***	**0.02***
Neutrophils ≥4% in BAL, n(%)	5 (27.8%)	10 (37%)	14 (48.3%)	0.26	0.07	0.2
**Bronchoalveolar lavage fluid biomarkers†**						
Periostin, ng/ml	0.75 (0.72-0.95)	0.96 (0.79-1.1)	0.84 (0.81-0.9)	0.35	0.99	0.49
Interleukin-6, pg/ml	0.79 (0.24-1.26)	0.7 (0.2-1.1)	0.68 (0.005-1.1)	0.99	0.99	0.99
Interleukin-12(p70), pg/ml	0.26 (0.06-0.42)	0.07 (0.06-0.12)	0.06 (0.04-0.09)	0.22	**0.006***	0.43
**Asthma therapy**						
Oral corticosteroids	0 (0%)	0 (0%)	14 (48%)	0.5	**<0.001***	**<0.001***
Inhaled corticosteroids (persistent use)	12 (46%)	27 (100%)	29 (100%)	**<0.001***	**<0.001***	1
Long-acting β_2-agonists (persistent use)	0 (0%)	25 (93%)	29 (100%)	**<0.001***	**<0.001***	0.15
Antileukotrienes	2 (11.1%)	3 (11%)	5 (17%)	0.5	0.29	0.26
Theophylline	0 (0%)	2 (7%)	6 (21%)	0.13	**0.04***	0.07
Long-acting anticholinergics (persistent use)	0 (0%)	0 (0%)	5 (17.%)	0.5	**0.03***	**0.01***

Categorical variables are presented as numbers (percentages), continuous variables as median and 0.25-0.75 quartiles, or mean and standard deviation, as appropriate. Abbreviations and references: BAL – bronchoalveolar lavage fluid, FEV_1 - forced expiratory volume in one second, GINA - Global Initiative for Asthma, L-liter, VC - vital capacity, § – asthma symptom control (assessed based on Asthma Control Test results), # - BAL cell differential data available in 67 asthma

subjects, † - BAL fluid levels of interleukin (IL)-4, IL-5, IL-10, IL-17A, and interferon γ were below the detection threshold (data not shown), ¥- RBM available in 45 asthma subjects, *- $p<0.05$.
